# A Physio-Logging Journey: Heart Rates of the Emperor Penguin and Blue Whale

**DOI:** 10.3389/fphys.2021.721381

**Published:** 2021-08-03

**Authors:** Paul J. Ponganis

**Affiliations:** Scripps Institution of Oceanography, University of California San Diego, La Jolla, CA, United States

**Keywords:** bio-logging, blue whale, cetacean, dive response, electrocardiogram, emperor penguin, heart rate, pinniped

## Abstract

Physio-logging has the potential to explore the processes that underlie the dive behavior and ecology of marine mammals and seabirds, as well as evaluate their adaptability to environmental change and other stressors. Regulation of heart rate lies at the core of the physiological processes that determine dive capacity and performance. The bio-logging of heart rate in unrestrained animals diving at sea was infeasible, even unimaginable in the mid-1970s. To provide a historical perspective, I review my 40-year experience in the development of heart rate physio-loggers and the evolution of a digital electrocardiogram (ECG) recorder that is still in use today. I highlight documentation of the ECG and the interpretation of heart rate profiles in the largest of avian and mammalian divers, the emperor penguin and blue whale.

## Introduction

The diving prowess of marine mammals and seabirds has long fascinated biologists as well as the lay public. Routine durations and depths of deep dives of exceptional divers are remarkable: 60–70 min and 1,400 m in Cuvier’s beaked whale (*Ziphius cavirostris*), 20–30 min and 400–600 m in elephant seals (*Mirounga* sp.), and 8–12 min and 400–500 m in emperor penguins (*Aptenodytes forsteri*) (Le Boeuf et al., 1986; Hindell et al., 1991; Kooyman and Kooyman, 1995; Tyack et al., 2006; Sato et al., 2011; Robinson et al., 2012; Schorr et al., 2014; Shearer et al., 2019; Kooyman et al., 2020). Maximum reported dive durations are even more impressive: 3.7 h in Cuvier’s beaked whale, 2 h in elephant seals, and 32 min in emperor penguins (Hindell et al., 1991; Stewart and DeLong, 1995; Goetz et al., 2018; Quick et al., 2020).

Such dive performance is dependent on increased oxygen (O_2_) storage, hypoxemic tolerance, pressure tolerance, and the regulation of metabolism (Ponganis, 2015). The latter is achieved through the interplay of cardiovascular responses, thermoregulation, body size, hydrodynamics, and cost-efficient swimming (Kooyman and Ponganis, 1998; Davis, 2014; Williams and Davis, 2021). The dive response, which consists of the breath hold (apnea), a decrease in heart rate (bradycardia), and peripheral vasoconstriction, underlies the management of O_2_ stores and organ O_2_ consumption through the regulation of the magnitude and distribution of tissue blood flow (Ponganis et al., 2011; Panneton, 2013; Panneton and Gan, 2020).

The intensity of the cardiovascular dive response (the degree of bradycardia and vasoconstriction during the breath hold) has long been known to be variable, even during the extreme bradycardias of the early forced submersion experiments of Scholander and Irving (Irving et al., 1941a,b; Grinnell et al., 1942; Irving et al., 1942; Jones et al., 1973; Butler, 1982; Jobsis et al., 2001). Heart rate profiles during dives in the wild have revealed that a variable, and often, moderate bradycardia occurred in many species, including gray seals (*Halichoerus grypus*), Weddell seals (*Leptonychotes weddellii*), elephant seals, Antarctic fur seals (*Arctocephalus gazella*), California sea lions (*Zalophus californianus*), narwhals (*Monodon monoceros*), blue whales (*Balaenoptera musculus*), South Georgian shags (*Phalacrocorax atriceps georgianus*), king penguins (*A. patagonicus*), and emperor penguins (Kooyman and Campbell, 1972; Hill et al., 1987; Thompson and Fedak, 1993; Andrews et al., 1997; Bevan et al., 1997; Hindell and Lea, 1998; Boyd et al., 1999; Froget et al., 2004; McDonald and Ponganis, 2014; Wright et al., 2014; Williams et al., 2017; Goldbogen et al., 2019). Recent research has emphasized the potential effects of exercise, depth, and volitional control on modulation of the bradycardia during dives (Davis and Williams, 2012; Noren et al., 2012; Williams et al., 2015; Elmegaard et al., 2016; Ponganis et al., 2017; Elmegaard et al., 2019). Although available evidence indicates that digestion and adequate renal/hepatic function were maintained during short duration, aerobic dives of Weddell seals (Davis et al., 1983; Davis, 2014), examination of simultaneous heart rate, and organ blood flow responses during dives in the wild have not been performed. Muscle blood flow, as inferred from muscle myoglobin saturation profiles, appeared variable both within and among dives of Weddell seals and emperor penguins (Guyton et al., 1995; Williams et al., 2011).

Given the importance of heart rate in the physiology and duration of a dive, and as a contribution to the history of physio-logging in the inauguration of *Physio-logging* in *Frontiers*, the editors have asked me to review my experience in the development of an electrocardiogram (ECG) logger. To convey the advances over the past 40 years to readers, I begin with the state of heart rate records in free-diving animals in the late 1970s. I then progress through the use of various types of recorders during my collaborations with Dr. Jerry Kooyman in the 1980s and 1990s to the eventual development in the early 2000s of an ECG logger that is still in use today. I conclude with the application of that logger to the largest avian and mammalian divers, the emperor penguin and the blue whale.

This mini-review is not intended to be a comprehensive examination of diving physiology or of the development and application of various physio-logging devices and techniques. For such information, readers are referred to prior publications (Butler and Jones, 1997; Kooyman and Ponganis, 1998; Weimerskirch et al., 2000; Ropert-Coudert et al., 2006; Ponganis, 2007; Sakamoto et al., 2013; Davis, 2014; Williams and Ponganis, 2021; Williams and Hindle, 2021). In those reviews and papers, the works of Butler and Woakes, Kanwisher, Fedak, Hill and Zapol, Andrews and Jones, Weimerkirsch, Ropert-Coudert, K. Sakamoto, T. Williams, and M. Johnson are relevant to the development of heart rate physio-logging techniques and recorders in many different marine mammals and seabirds.

## State of the Art in the 1970s

Prior to 1980, most heart rate records during unrestrained breath holds and diving in marine mammals were obtained in animals under managed care with use of bench top ECG recorders attached to long leads or with use of radiotelemetry. Investigations included sea lions, harbor seals (*Phoca vitulina*), gray seals, dolphins, a beluga (*Delphinapterus leucas*), a killer whale (*Orcinus orca*), and even a non-marine mammal, the hippopotamus (*Hippopotamus amphibius*) (Irving et al., 1941a; King et al., 1953; Elsner, 1965, 1966; Elsner et al., 1966; Spencer et al., 1967; Ridgway, 1972; Jones et al., 1973; Ridgway et al., 1975a,b; Kanwisher and Ridgway, 1983). Apart from an ECG of a harpooned beluga (King et al., 1953; White et al., 1953), the only ECG and heart rate data from a wild animal free-diving at sea were partial records obtained in Weddell seals with long, break-away ECG electrodes, and a bench top recorder (Kooyman and Campbell, 1972).

Telemetry research on unrestrained, spontaneous dives of birds at this time primarily focused on ducks in laboratory tanks (Butler and Woakes, 1979; Butler, 1982). Heart rate profiles of hand-reared cormorants diving in a bay were obtained with the use of acoustic telemetry (Kanwisher et al., 1981). In Humboldt penguins (*Spheniscus humboldti*), radiotelemetry transmitters documented heart rates during dives of up to 50-s duration in a laboratory tank (Butler and Woakes, 1984). In the only cardiovascular study of diving birds in the wild during this time period, heart rate was obtained with telemetered arterial blood velocity profiles from a tethered gentoo penguin (*Pygoscelis papua*) spontaneously diving in the sea (Millard et al., 1973).

In summary, heart rate profiles during spontaneous breath holds and dives of marine mammals and birds in this era were limited to short duration, shallow dives usually in laboratory tanks or pools. The longest published recordings were Kooyman’s study of the Weddell seal with break-away ECG leads at an isolated dive hole on the sea ice of McMurdo Sound, Antarctica (Kooyman and Campbell, 1972). Heart rates were successfully recorded during resting/slow swimming shallow dives beneath the ice for up to 5 min. Initial heart rates during deep dives were only recorded for 30–45 s due to a maximum length of 70 m for the ECG leads.

## 1980s–1990s: an Early Heart Rate Logger and an Underwater Holter Monitor

As a medical student and anesthesiology resident at Stanford in the late 1970s and early 1980s, I was acquainted with the Holter monitor, a medical device that recorded the ECG continuously on cassette tape for 24–48 h in ambulatory patients (Del Mar, 2005). I wondered how this monitor could be applied to a diving seal. The unit would have to be protected in a waterproof housing, but a depth recorder was also needed. At the time, the most advanced time depth recorder (TDR) utilized a light emitting diode (LED) that transcribed the depth profile onto a scrolled roll of film (Kooyman et al., 1976). In talking about this idea with my brother, Ed, an electrical engineer, he suggested that this could all be recorded electronically, both the depth and the heart rate. And, furthermore, he could make it. I was amazed. He began to build an electronic heart rate/depth recorder in his spare time. In 1982, we began a collaboration with Kooyman for testing and further development.

Our efforts eventually resulted in a physio-logger that could record depth, swim velocity, heart rate (number of heart beats in a 15-s period counted with a built-in detector for the ECG signal), temperature (from a thermistor), and the partial pressure of oxygen (from an intravascular electrode which proved too fragile and susceptible to shorting by saltwater). The ECG signal was recognized by detection of the R wave, the positive wave associated with ventricular contraction. Computerized processing and analyses of the data were conducted by my wife, Dr. Katherine Ponganis, a cosmochemist and computer expert, who brought Kooyman and me into the computer age. In the 1987–1988 Antarctic field season, we successfully obtained heart rates and swim speeds of emperor penguins diving at an isolated dive hole (Kooyman et al., 1992).

This physio-logger was limited by memory capacity, only 5 h with heart rate recorded at 15-s intervals and depth every 4 s. Furthermore, counting of high heart rates above 100 beats min^−1^ (bpm) was limited by the R-wave detector, which had a programmed refractory period to prevent the erroneous counting of T waves (the positive waveforms associated with ventricular repolarization in the ECG signal) as R waves. Re-programming of the microprocessor to record the ECG signal at 250 Hz confirmed the accurate detection of heart rates during the dive and demonstrated that heart rates at the surface were greater than the 100-bpm limitation. However, in the 250-Hz mode, memory capacity was only 20 min.

Based on the limitations of that physio-logger, and the variable shapes/sizes of ECG signals recorded with surface electrodes in different species, we decided to return to the Holter monitor to record the actual ECG signal in continuing studies. Rather than reliance on an R-wave detector to recognize the R wave and count the heart beats, heart rate would be calculated on a beat-to-beat basis from the digitized ECG record. One disadvantage of the Holter monitor was the need to use a proprietary ECG processor to print out the analogue ECG record. Fortunately, my colleagues at the San Diego Cardiac Center were happy to help. Another limitation was size. Even with the smallest available Holter monitor at the time (SpaceLabs, Inc., Model 90205), the entire unit, including the underwater housing, weighed 1 kg. Nonetheless, the Holter monitor was successfully applied to three species: young California sea lions trained to dive at sea and swim underwater in the ring tank at Scripps, Lake Baikal seals (*P. sibirica*) during spontaneous submersions (up to 25-min duration) in their lakeside tanks at Listvyanka, and a rehabilitated gray whale calf (*Eschrichtius robustus*) at SeaWorld prior to its release (Ponganis et al., 1997a,b; Ponganis and Kooyman, 1999). The same model Holter monitor was also successfully used with diving northern elephant seals during this time period (Andrews et al., 1997).

### The Early 2000s and Beyond: A Digital ECG Recorder

On return to cardiovascular research on emperor penguins in the early 2000s, I needed a small, backpack-type digital ECG recorder to provide a continuous ECG record from which heart rate could be calculated on a beat-to-beat basis. Obviously, the Holter monitor was too large. And digital ECG recorders developed for seals were not available (Hill, 1986; Andrews, 1998). So, I turned to the late Harve Hanish, engineer and owner of UFI (Morro Bay, CA), who was already making temperature recorders for my research. Harve’s curiosity, engineering skills, and interest in promoting scientific research were exemplar. My association with him and UFI led to the development of physio-loggers for temperature, ECG, intravascular P_O2_, and near-infrared muscle myoglobin saturation as well as to the construction of a backpack blood sampler (Ponganis et al., 2003; Stockard et al., 2005; Ponganis et al., 2007; Meir et al., 2008, 2009; Meir and Ponganis, 2009; Ponganis et al., 2009; Meir and Ponganis, 2010; Williams et al., 2011; Williams and Hicks, 2016; Williams et al., 2021a). Although most of these commercially available recorders have only been used by my research group, other researchers have applied various versions of this ECG recorder to bottlenose dolphins (*Tursiops truncatus*), Weddell seals, and narwhals (Davis and Williams, 2012; Williams et al., 2015, 2017).

The development of the ECG logger was not the only hurdle to document heart rate, however. A custom ECG peak detection – heart rate program designed by Dr. Katherine Ponganis in the early 2000s was fundamental and has formed the basis of many a paper by graduate students and postdoctoral researchers. The underwater housings, which never leaked, were built by the late Jim Billups (Meer Instruments, San Diego CA). He had been Kooyman’s engineer for the LED-film TDRs in the 1970s. In addition, different types of electrodes (skin surface, subcutaneous, intravascular, and suction cup) had to be developed by trial and error. Suction cup electrodes and the suction cup – float attachment for cetaceans evolved over 3 years (2015–2018) with the advice of Drs. Mark Johnson, Ari Friedlaender, and Jeremy Goldbogen, and with the cooperation of Customized Animal Tracking Solutions.^1^ Collaboration with SeaWorld of San Diego was essential to the evaluation and positioning of the suction cup electrodes for cetaceans.

In my work with multiple collaborators, this ECG recorder has proved quite versatile and has been used to document heart rates in emperor penguins (both at an isolated dive hole camp and at sea), California sea lions at sea, loggerhead sea turtles (*Caretta caretta*), blue whales, trained bottlenose dolphins, pilot whales (*Globicephala macrorhynchus*), belugas, and killer whales (Meir et al., 2008; Houser et al., 2010; McDonald and Ponganis, 2014; Wright et al., 2014; Bickett et al., 2019; Goldbogen et al., 2019; Williams et al., 2019; McDonald et al., 2020). I will now highlight heart rate findings in the largest avian and mammalian divers, the emperor penguin and blue whale.

### Emperor Penguins and Blue Whales

**Figure 1:**
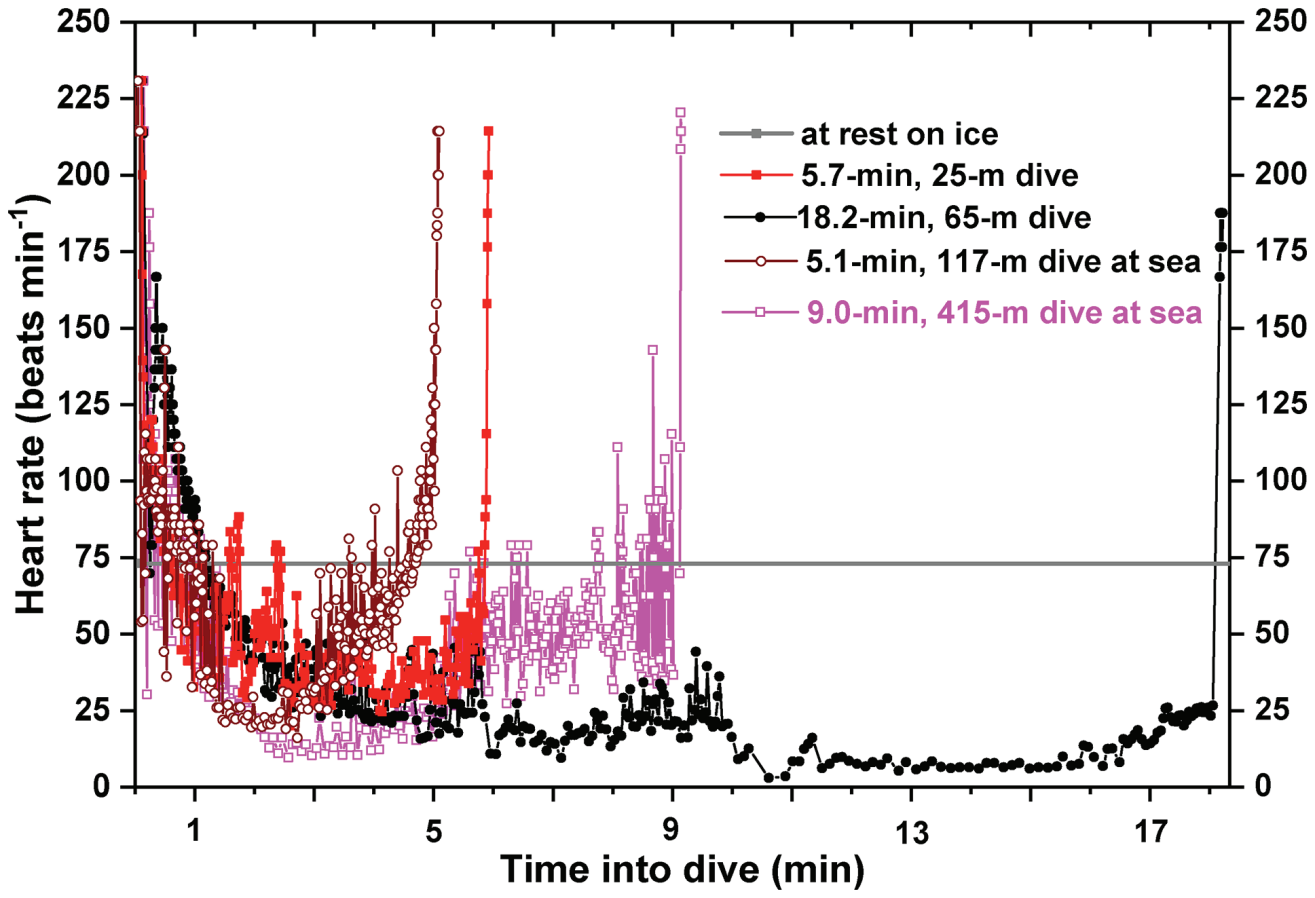
Beat-to-beat heart rate profiles of emperor penguins diving either at an experimental isolated dive hole in McMurdo Sound, Antarctica, or during foraging trips at sea from Cape Washington, Antarctica. Heart rate profiles were characterized by an immediate decline from high pre-dive levels [200–240 beats min^−1^ (bpm)], a continued gradual decline to below-resting levels over the initial 1 to 2 min, and then a further progressive decline as dive duration increased. In the bottom phase of long dives and deep dives, heart rates could be as low as 5–10 bpm. Ascents were characterized by a gradual increase in heart rate. Heart rate profiles were characterized by abrupt oscillations in heart rate throughout the dive. Heart rate at rest was about 72 bpm. Adapted from the data of Meir et al. (2008) and Wright et al. (2014).

In emperor penguins, beat-to-beat heart rate profiles obtained from the digital ECG recorder during dives (Figure 1) reflected the averaged 15-s heart rate profiles found in the earlier study (Kooyman et al., 1992) and, importantly, demonstrated the variability and control of heart rate in exquisite detail. Heart rates declined from high inter-dive surface values, initially hovered at near-resting and even below-resting values, and then continued to decrease, sometimes to values as low as 5–10 beats min^−1^ (bpm), as dives became longer and deeper (Meir et al., 2008; Wright et al., 2014). During ascent, heart rate increased as in other species (Figure 1). Dive heart rate (total number of beats/dive duration) was typically above the resting level for short duration dives, but it progressively decreased as dive duration increased. The dive response of emperor penguins was variable both in the intensity of bradycardia and the degree and pattern of vasoconstriction as evidenced by muscle myoglobin desaturation profiles during dives (Williams et al., 2011). During the bottom phases of 400–500-m deep dives, heart rates were lowest while wing stroke rates were usually quite high (Williams et al., 2012; Wright et al., 2014). In these long deep dives, heart rate appeared uncoupled from exercise intensity. More recent papers have also found plasticity in the dive response and have evaluated the potential effects of exercise on heart rate in different types of dives of seals, sea lions, and cetaceans (Davis and Williams, 2012; Noren et al., 2012; McDonald and Ponganis, 2014; Williams et al., 2015, 2017; McDonald et al., 2018).

**Figure 2:**
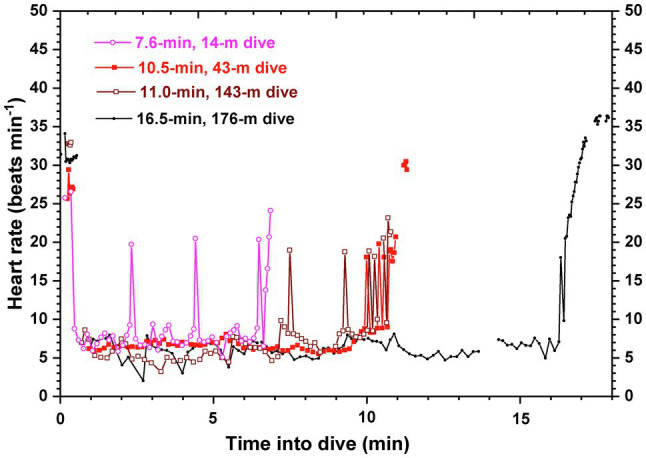
Beat-to-beat heart rate profiles of four dives of a blue whale in Monterey Bay, CA. Heart rates were typically 4–8 beats min^−1^ (bpm) during the bottom phases of both shallow and deep dives. Oscillations in heart rate were not uncommon. Pre- and post-dive heart rates after deep dives were near 35 bpm; for shallow, short duration dives, surface heart rates were typically 25–30 bpm. The allometrically predicted resting heart rate of a 70,000 kg blue whale was 15 bpm. Gaps in the heart rate profile were due to artifact in the ECG record. Adapted from the data of Goldbogen et al. (2019).

The first successful deployment of an ECG recorder on a large whale without prior restraint or capture occurred in 2018. In the blue whale, beat-to-beat heart rate profiles during and between dives at sea (Figure 2) confirmed a variable and intense dive response during which heart rates were routinely 4–8 bpm, well below the predicted resting heart rate of 15 bpm for a 70,000-kg animal (Goldbogen et al., 2019). Furthermore, based on the time required for the contraction-relaxation cycle of a single heartbeat (estimated from the ECG signal), surface interval heart rates between deep dives were near maximal. A diving bradycardia underlied O_2_ store management and conserved blood O_2_ even in an animal as large and with as low a predicted resting heart rate as the blue whale. The low heart rates during dives were also consistent with biomechanical and anatomical analyses of hemodynamic function in the whale aorta (Shadwick and Gosline, 1994). At such low heart rates during dives, the compliant, elastic aortic arch of the whale acts as a windkessel to maintain blood pressure and blood flow during the long pause between such slow heart beats. In addition, at the higher heart rates at the surface, the pressure wavelengths allow for destructive interference of outgoing and reflected pressure waves in the aorta, thus decreasing the impedance against which the heart must pump.

## Summary

At the start of my bio-logging career 40 years ago, the physio-logging of heart rate in unrestrained marine mammals and seabirds diving at sea was infeasible, even unimaginable. The evolution of a digital ECG recorder has involved improvements/miniaturization in electrodes, recorder design and memory capacity, battery life, pressure-proof underwater housings, and attachment techniques. The initial evaluation of prototype recorders with animals under permanent or temporary managed care has been invaluable.

Despite such progress in the physio-logging of heart rate in marine mammals and seabirds, there is still room for improvement in the future. Movement artifact and diminution of the ECG signal by saltwater may be prevented or minimized with further electrode modifications and use of capacitative ECG electrodes (Ha et al., 2014; Reyes et al., 2014; Thap et al., 2016; Tripathi et al., 2017). In addition, detection of heartbeats may be achieved with different noninvasive approaches, including acoustics, near-infrared spectroscopy, photoplethysmography, and nanotechnology (Burgess et al., 1998; McKnight et al., 2019; dos Santos et al., 2021; Williams et al., 2021b). Equally important is the continued refinement of data processing and analysis.

Continued refinement of the bio-logging of heart rate and other cardiovascular parameters will provide a better understanding of the dive response and its role in O_2_ store management, the uptake and distribution of nitrogen (risk of decompression sickness), and thermoregulation. As such, physio-logging has the potential not only to explore the processes that underlie the dive behavior and ecology of these species, but also to evaluate their adaptability to environmental change and other stressors.

## Author Contributions

PP conceived and wrote this paper. The author confirms being the sole contributor of this work and has approved it for publication.

## Conflict of Interest

The author declares that the research was conducted in the absence of any commercial or financial relationships that could be construed as a potential conflict of interest.

## Publisher’s Note

All claims expressed in this article are solely those of the authors and do not necessarily represent those of their affiliated organizations, or those of the publisher, the editors and the reviewers. Any product that may be evaluated in this article, or claim that may be made by its manufacturer, is not guaranteed or endorsed by the publisher.

## Footnotes

^1^ www.cats.is

## References

[ref1] Andrews R. D. (1998). Instrumentation for the remote monitoring of physiological and behavioral variables. J. Appl. Physiol. 85, 1974–1981. 10.1152/jappl.1998.85.5.1974, PMID: 9804606

[ref2] Andrews R. D. Jones D. R. Williams J. D. Thorson P. H. Oliver G. W. Costa D. P.. (1997). Heart rates of northern elephant seals diving at sea and resting on the beach. J. Exp. Biol. 200, 2083–2095. 10.1242/jeb.200.15.2083, PMID: 9255950

[ref3] Bevan R. M. Boyd I. L. Butler P. J. Reid K. R. Woakes A. J. Croxall J. P. (1997). Heart rates and abdominal temperatures of free-ranging south Georgian shags, *Phalacrocorax georgianus*. J. Exp. Biol. 200, 661–675. 10.1242/jeb.200.4.661, PMID: 9318399

[ref4] Bickett N. J. Tift M. S. St. Leger J. Ponganis P. J. (2019). Heart rates, heart rate profiles, and electrocardiograms in three killer whales, a beluga, and a pilot whale: an exploratory investigation. Mar. Mamm. Sci. 35, 1112–1132. 10.1111/mms.12578

[ref5] Boyd I. L. Bevan R. M. Woakes A. J. Butler P. J. (1999). Heart rate and behavior of fur seals: implications for measurement of field energetics. Am. J. Phys. 276, H844–H857. 10.1152/ajpheart.1999.276.3.H844, PMID: 10070067

[ref6] Burgess W. C. Tyack P. L. LeBoeuf B. J. Costa D. P. (1998). A programmable acoustic recording tag and first results from free-ranging northern elephant seals. Deep-Sea Res. II 45, 1327–1351. 10.1016/S0967-0645(98)00032-0

[ref7] Butler P. J. (1982). Respiratory and cardiovascular control during diving in birds and mammals. J. Exp. Biol. 100, 195–221. 10.1242/jeb.100.1.195, PMID: 6757368

[ref8] Butler P. J. Jones D. R. (1997). The physiology of diving of birds and mammals. Physiol. Rev. 77, 837–899. 10.1152/physrev.1997.77.3.837, PMID: 9234967

[ref9] Butler P. J. Woakes A. J. (1979). Changes in heart rate and respiratory frequency during natural behaviour of ducks, with particular reference to diving. J. Exp. Biol. 79, 283–300. 10.1242/jeb.79.1.283

[ref10] Butler P. J. Woakes A. J. (1984). Heart rate and aerobic metabolism in Humboldt penguins (*Spheniscus humboldti*) during voluntary dives. J. Exp. Biol. 108, 419–428. 10.1242/jeb.108.1.419, PMID: 6423763

[ref11] Davis R. W. (2014). A review of the multi-level adaptations for maximizing aerobic dive duration in marine mammals: from biochemistry to behavior. J. Comp. Physiol. B 184, 23–53. 10.1007/s00360-013-0782-z, PMID: 24126963

[ref12] Davis R. W. Castellini M. A. Kooyman G. L. Maue R. (1983). Renal GFR and hepatic blood flow during voluntary diving in Weddell seals. Am. J. Phys. 245, R743–R748. 10.1152/ajpregu.1983.245.5.R743, PMID: 6638219

[ref13] Davis R. W. Williams T. M. (2012). The marine mammal dive response is exercise modulated to maximize aerobic dive duration. J. Comp. Physiol. A 198, 583–591. 10.1007/s00359-012-0731-4, PMID: 22585422

[ref14] Del Mar B. (2005). The history of clinical Holter monitoring. Ann. Noninvasive Electrocardiol. 10, 226–230. 10.1111/j.1542-474X.2005.10202.x, PMID: 15842436 PMC6932614

[ref15] dos Santos C. C. Lucena G. N. Pinto G. C. Júnior M. J. Marques R. F. C. (2021). Advances and current challenges in non-invasive wearable sensors and wearable biosensors—A mini-review. Med. Devices Sens. 4:e10130. 10.1002/mds3.10130

[ref16] Elmegaard S. E. Johnson M. Madsen P. T. McDonald B. I. (2016). Cognitive control of heart rate in diving harbor porpoises. Curr. Biol. 26, R1167–R1176. 10.1016/j.cub.2016.10.020, PMID: 27875692

[ref17] Elmegaard S. L. McDonald B. I. Madsen P. T. (2019). Drivers of the dive response in trained harbour porpoises (*Phocoena phocoena*). J. Exp. Biol. 222:jeb208637. 10.1242/jeb.208637, PMID: 31511341

[ref18] Elsner R. (1965). Heart rate response in forced versus trained experimental dives of pinnipeds. Hvalradets Skrifter 48, 24–29.

[ref19] Elsner R. (1966). Diving bradycardia in the unrestrained hippopotamus. Nature 212:408. 10.1038/212408a0, PMID: 5970156

[ref20] Elsner R. Kenney D. W. Burgess K. (1966). Bradycardia in the trained dolphin. Nature 212:407. 10.1038/212407a0, PMID: 5970155

[ref21] Froget G. Butler P. J. Woakes A. J. Fahlman A. Kuntz G. Le Maho Y.. (2004). Heart rate and energetics of free-ranging king penguins (*Aptenodytes patagonicus*). J. Exp. Biol. 207, 3917–3926. 10.1242/jeb.01232, PMID: 15472022

[ref22] Goetz K. T. McDonald B. I. Kooyman G. L. (2018). Habitat preference and dive behavior of non-breeding emperor penguins in the eastern Ross Sea, Antarctica. Mar. Ecol. Prog. Ser. 593, 155–171. 10.3354/meps12486

[ref23] Goldbogen J. A. Cade D. E. Calambokidis J. Czapanskiy M. F. Fahlbusch J. Friedlaender A. S.. (2019). Extreme bradycardia and tachycardia in the world’s largest animal. Proc. Natl. Acad. Sci. 116, 25329–25332. 10.1073/pnas.1914273116, PMID: 31767746 PMC6911174

[ref24] Grinnell S. W. Irving L. Scholander P. F. (1942). Experiments on the relation between blood flow and heart rate in the living seal. J. Cell. Comp. Physiol. 19, 341–350. 10.1002/jcp.1030190309

[ref25] Guyton G. P. Stanek K. S. Schneider R. C. Hochachka P. W. Hurford W. E. Zapol D. G.. (1995). Myoglobin-saturation in free-diving Weddell seals. J. Appl. Physiol. 79, 1148–1155. 10.1152/jappl.1995.79.4.1148, PMID: 8567556

[ref26] Ha S. Kim C. Chi Y. M. Akinin A. Maier C. Ueno A.. (2014). Integrated circuits and electrode interfaces for noninvasive physiological monitoring. IEEE Trans. Biomed. Eng. 61, 1522–1537. 10.1109/TBME.2014.2308552, PMID: 24759282

[ref27] Hill R. D. (1986). Microcomputer monitor and blood sampler for free-diving Weddell seals *Leptonychotes weddelli*. J. Appl. Physiol. 61, 1570–1576. 10.1152/jappl.1986.61.4.1570, PMID: 3781968

[ref28] Hill R. D. Schneider R. C. Liggins G. C. Schuette A. H. Elliott R. L. Guppy M.. (1987). Heart rate and body temperature during free diving of Weddell seals. Am. J. Phys. 253, R344–R351. 10.1152/ajpregu.1987.253.2.R344, PMID: 3618833

[ref29] Hindell M. A. Lea M. A. (1998). Heart rate, swimming speed, and estimated oxygen consumption of a free-ranging southern elephant seal. Physiol. Zool. 71, 74–84. 10.1086/515890, PMID: 9472815

[ref30] Hindell M. A. Slip D. J. Burton H. R. (1991). The diving behaviour of adult male and female southern elephant seals, *Mirounga leonina* (Pinnipedia: Phocidae). Aust. J. Zool. 39, 595–619. 10.1071/ZO9910595

[ref31] Houser D. S. Dankiewicz-Talmadge L. A. Stockard T. K. Ponganis P. J. (2010). Investigation of the potential for vascular bubble formation in a repetitively diving dolphin. J. Exp. Biol. 213, 52–62. 10.1242/jeb.028365, PMID: 20008362

[ref32] Irving L. Scholander P. F. Grinnell S. W. (1941a). The respiration of the porpoise, *Tursiops truncatus*. J. Cell. Comp. Physiol. 17, 145–168. 10.1002/jcp.1030170203

[ref33] Irving L. Scholander P. F. Grinnell S. W. (1941b). Significance of the heart rate to the diving ability of seals. J. Cell. Comp. Physiol. 18, 283–297. 10.1002/jcp.1030180302

[ref34] Irving L. Scholander P. F. Grinnell S. W. (1942). The regulation of arterial blood pressure in the seal during diving. Am. J. Phys. 135, 557–566. 10.1152/ajplegacy.1942.135.3.557

[ref35] Jobsis P. D. Ponganis P. J. Kooyman G. L. (2001). Effects of training on forced submersion responses in harbor seals. J. Exp. Biol. 204, 3877–3885. 10.1242/jeb.204.22.3877 11807105

[ref36] Jones D. R. Fisher H. D. McTaggart S. West N. H. (1973). Heart rate during breath-holding and diving in the unrestrained harbor seal (*Phoca vitulina richardi*). Can. J. Zool. 51, 671–680. 10.1139/z73-101, PMID: 4756147

[ref37] Kanwisher J. W. Gabrielsen G. Kanwisher N. (1981). Free and forced diving in birds. Science 211, 717–719. 10.1126/science.7192883, PMID: 7192883

[ref38] Kanwisher J. W. Ridgway S. H. (1983). The physiological ecology of whales and porpoises. Sci. Am. 248, 110–120. 10.1038/scientificamerican0683-110

[ref39] King R. L. Jenks J. L. White P. D. (1953). The electrocardiogram of a beluga whale. Circulation 8, 387–393. 10.1161/01.CIR.8.3.387, PMID: 13082672

[ref40] Kooyman G. L. Campbell W. B. (1972). Heart rates in freely diving Weddell seals, *Leptonychotes weddelli*. Comp. Biochem. Physiol. A Physiol. 43, 31–36. 10.1016/0300-9629(72)90465-3, PMID: 4404585

[ref41] Kooyman G. L. Gentry R. L. Urquhart D. L. (1976). Northern fur seal diving behavior: a new approach to its study. Science 193, 411–412. 10.1126/science.935876, PMID: 935876

[ref42] Kooyman G. L. Goetz K. Williams C. L. Ponganis P. J. Sato K. Eckert S.. (2020). Crary bank: a deep foraging habitat for emperor penguins in the western Ross Sea. Polar Biol. 43, 801–811. 10.1007/s00300-020-02686-3

[ref43] Kooyman G. L. Kooyman T. G. (1995). Diving behavior of emperor penguins nurturing chicks at Coulman Island, Antarctica. Condor 97, 536–549. 10.2307/1369039

[ref44] Kooyman G. L. Ponganis P. J. (1998). The physiological basis of diving to depth: birds and mammals. Annu. Rev. Physiol. 60, 19–32. 10.1146/annurev.physiol.60.1.19, PMID: 9558452

[ref45] Kooyman G. L. Ponganis P. J. Castellini M. A. Ponganis E. P. Ponganis K. V. Thorson P. H.. (1992). Heart rates and swim speeds of emperor penguins diving under sea ice. J. Exp. Biol. 165, 161–180. 10.1242/jeb.165.1.161, PMID: 1588249

[ref46] Le Boeuf B. J. Costa D. P. Huntley A. C. Kooyman G. L. Davis R. W. (1986). Pattern and depth of dives in northern elephant seals, *Mirounga angustirostris*. J. Zool. 208, 1–7. 10.1111/j.1469-7998.1986.tb04705.x

[ref47] McDonald B. I. Johnson M. Madsen P. T. (2018). Dive heart rate in harbour porpoises is influenced by exercise and expectations. J. Exp. Biol. 221:jeb168740. 10.1242/jeb.168740, PMID: 29122951

[ref48] McDonald B. I. Ponganis P. J. (2014). Deep-Diving Sea lions exhibit extreme bradycardia in long-duration dives. J. Exp. Biol. 217, 1525–1534. 10.1242/jeb.098558, PMID: 24790100

[ref49] McDonald B. I. Tift M. S. Hückstädt L. A. Jeffko M. Ponganis P. J. (2020). Stroke effort and relative lung volume influence heart rate in diving sea lions. J. Exp. Biol. 223:jeb214163. 10.1242/jeb.214163, PMID: 32098880

[ref50] McKnight J. C. Bennett K. A. Bronkhorst M. Russell D. J. F. Balfour S. Milne R.. (2019). Shining new light on mammalian diving physiology using wearable near-infrared spectroscopy. PLoS Biol. 17:e3000306. 10.1371/journal.pbio.3000306, PMID: 31211787 PMC6581238

[ref51] Meir J. U. Champagne C. D. Costa D. P. Williams C. L. Ponganis P. J. (2009). Extreme hypoxemic tolerance and blood oxygen depletion in diving elephant seals. Am. J. Physiol. Regul. Integr. Comp. Physiol. 297, R927–R939. 10.1152/ajpregu.00247.2009, PMID: 19641132

[ref52] Meir J. U. Ponganis P. J. (2009). High-affinity hemoglobin and blood oxygen saturation in diving emperor penguins. J. Exp. Biol. 212, 3330–3338. 10.1242/jeb.033761, PMID: 19801437

[ref53] Meir J. U. Ponganis P. J. (2010). Blood temperature profiles of diving elephant seals. Physiol. Biochem. Zool. 83, 531–540. 10.1086/651070, PMID: 20334547

[ref54] Meir J. U. Stockard T. K. Williams C. L. Ponganis K. V. Ponganis P. J. (2008). Heart rate regulation and extreme bradycardia in diving emperor penguins. J. Exp. Biol. 211, 1169–1179. 10.1242/jeb.013235, PMID: 18375841

[ref55] Millard R. W. Johansen K. Milsom W. K. (1973). Radiotelemetry of cardiovascular responses to exercise and diving in penguins. J. Comp. Biochem. Physiol. A 46, 227–240. 10.1016/0300-9629(73)90414-3, PMID: 4147892

[ref56] Noren S. R. Kendall T. Cuccurullo V. Williams T. M. (2012). The dive response redefined: underwater behavior influences cardiac variability in freely diving dolphins. J. Exp. Biol. 215, 2735–2741. 10.1242/jeb.069583, PMID: 22837445

[ref57] Panneton W. M. (2013). The mammallian diving response: an enigmatic reflex to preserve life? Physiology 28, 284–297. 10.1152/physiol.00020.2013, PMID: 23997188 PMC3768097

[ref58] Panneton W. M. Gan Q. (2020). The mammalian diving response: inroads to its neural control. Front. Neurosci. 14:524. 10.3389/fnins.2020.00524, PMID: 32581683 PMC7290049

[ref59] Ponganis P. J. (2007). Bio-logging of physiological parameters in higher marine vertebrates. Deep-Sea Res. II 54, 183–192. 10.1016/j.dsr2.2006.11.009

[ref60] Ponganis P. J. (2015). Diving Physiology of Marine Mammals and Seabirds. Cornwall: Cambridge University Press.

[ref61] Ponganis P. J. Kooyman G. L. (1999). Heart rate and electrocardiogram characteristics of a young California gray whale (*Eschrictius robustus*). Mar. Mamm. Sci. 15, 1198–1207. 10.1111/j.1748-7692.1999.tb00885.x

[ref62] Ponganis P. J. Kooyman G. L. Baronov E. A. Thorson P. H. Stewart B. S. (1997a). The aerobic submersion limit of Baikal seals, *Phoca sibirica*. Can. J. Zool. 75, 1323–1327. 10.1139/z97-756

[ref63] Ponganis P. J. Kooyman G. L. Winter L. M. Starke L. N. (1997b). Heart rate and plasma lactate responses during submerged swimming and diving in California Sea lions (*Zalophus californianus*). J. Comp. Physiol. B 167, 9–16. 10.1007/s003600050042, PMID: 9051904

[ref64] Ponganis P. J. McDonald B. I. Tift M. S. Williams C. L. (2017). Heart rate regulation in diving sea lions: the vagus nerve rules. J. Exp. Biol. 220, 1372–1381. 10.1242/jeb.146779, PMID: 28424310

[ref65] Ponganis P. J. Meir J. U. Williams C. L. (2011). In pursuit of Irving and Scholander: a review of oxygen store management in seals and penguins. J. Exp. Biol. 214, 3325–3339. 10.1242/jeb.031252, PMID: 21957096

[ref66] Ponganis P. J. Stockard T. K. Meir J. U. Williams C. L. Ponganis K. V. Howard R. (2009). O_2_ store management in diving emperor penguins. J. Exp. Biol. 212, 217–224. 10.1242/jeb.026096, PMID: 19112140 PMC2720999

[ref67] Ponganis P. J. Stockard T. K. Meir J. U. Williams C. L. Ponganis K. V. van Dam R. P.. (2007). Returning on empty: extreme blood O_2_ depletion underlies dive capacity of emperor penguins. J. Exp. Biol. 210, 4279–4285. 10.1242/jeb.011221, PMID: 18055617

[ref68] Ponganis P. J. Van Dam R. P. Levenson D. H. Knower T. Ponganis K. V. Marshall G. (2003). Regional heterothermy and conservation of core temperature in emperor penguins diving under sea ice. Comp. Biochem. Physiol. A 135, 477–487. 10.1016/S1095-6433(03)00133-8, PMID: 12829055

[ref69] Quick N. J. Cioffi W. R. Shearer J. M. Fahlman A. Read A. J. (2020). Extreme diving in mammals: first estimates of behavioural aerobic dive limits in Cuvier’s beaked whales. J. Exp. Biol. 223:jeb222109. 10.1242/jeb.222109, PMID: 32967976

[ref70] Reyes B. A. Posada-Quintero H. F. Bales J. R. Clement A. L. Pins G. D. Swiston A.. (2014). Novel electrodes for underwater ECG monitoring. IEEE Trans. Biomed. Eng. 61, 1863–1876. 10.1109/TBME.2014.2309293, PMID: 24845297

[ref71] Ridgway S. H. (1972). Mammals of the Sea Biology and Medicine. Springfield: Charles C Thomas.

[ref72] Ridgway S. H. Carder D. A. Clark W. (1975a). Conditioned bradycardia in the sea lion *Zalophus californianus*. Nature 256, 37–38. 1134577 10.1038/256037a0

[ref73] Ridgway S. H. Harrison R. J. Joyce P. L. (1975b). Sleep and cardiac rhythm in the gray seal. Science 187, 553–555. 10.1126/science.163484, PMID: 163484

[ref74] Robinson P. W. Costa D. P. Crocker D. E. Gallo-Reynoso J. P. Champagne C. D. Fowler M. A.. (2012). Foraging behavior and success of a mesopelagic predator in the Northeast Pacific Ocean: insights from a data-rich species, the northern elephant seal. PLoS One 7:e36728. 10.1371/journal.pone.0036728, PMID: 22615801 PMC3352920

[ref75] Ropert-Coudert Y. Wilson R. P. Gremillet D. Kato A. Lewis S. Ryan P. G. (2006). Electrocardiogram recordings in free-ranging gannets reveal minimum difference in heart rate during flapping versus gliding flight. Mar. Ecol. Prog. Ser. 328, 275–284. 10.3354/meps328275

[ref76] Sakamoto K. Q. Takahashi A. Iwata T. Yamamoto T. Yamamoto M. Trathan P. N. (2013). Heart rate and estimated energy expenditure of flapping and gliding in black-browed albatrosses. J. Exp. Biol. 216, 3175–3182. 10.1242/jeb.079905, PMID: 23661772

[ref77] Sato K. Shiomi K. Marshall G. Kooyman G. L. Ponganis P. J. (2011). Stroke rates and diving air volumes of emperor penguins: implications for dive performance. J. Exp. Biol. 214, 2854–2863. 10.1242/jeb.055723, PMID: 21832128

[ref78] Schorr G. S. Falcone E. A. Moretti D. J. Andrews R. D. (2014). First long-term behavioral records from Cuvier’s beaked whales (*Ziphius cavirostris*) reveal record-breaking dives. PLoS One 26:e92633. 10.1371/journal.pone.0092633, PMID: 24670984 PMC3966784

[ref79] Shadwick R. E. Gosline J. M. (1994). Arterial mechanics in the fin whale suggest a unique hemodynamic design. Am. J. Phys. 267, R805–R818. 10.1152/ajpregu.1994.267.3.R805, PMID: 8092327

[ref80] Shearer J. Qiuick N. J. Cioffi W. R. Baird R. W. Webster D. L. Foley H. J.. (2019). Diving behaviour of Cuvier’s beaked whales (*Ziphius cavirostris*) off Cape Hatteras, North Carolina. R. Soc. Open Sci. 6:181728. 10.1098/rsos.181728, PMID: 30891284 PMC6408375

[ref81] Spencer M. P. Gornall T. A. Poulter T. C. (1967). Respiratory and cardiac activity of killer whales. J. Appl. Physiol. 22, 974–981. 10.1152/jappl.1967.22.5.974, PMID: 6025756

[ref82] Stewart B. S. DeLong R. L. (1995). Double migrations of the northern elephant seal, *Mirounga angustirostris*. J. Mammal. 76, 196–205. 10.2307/1382328

[ref83] Stockard T. K. Heil J. Meir J. U. Sato K. Ponganis K. V. Ponganis P. J. (2005). Air sac Po_2_ and oxygen depletion during dives of emperor penguins. J. Exp. Biol. 208, 2973–2981. 10.1242/jeb.01687, PMID: 16043602

[ref84] Thap T. Yoon K.-H. Lee J. (2016). Graphite based electrode for ECG monitoring: evaluation under freshwater and saltwater conditions. Sensors 16:542. 10.3390/s16040542, PMID: 27092502 PMC4851056

[ref85] Thompson D. Fedak M. A. (1993). Cardiac responses of grey seals during diving at sea. J. Exp. Biol. 174, 139–164. 10.1242/jeb.174.1.139, PMID: 8440964

[ref86] Tripathi R. P. Tiwari A. Mishra G. R. (2017). “Design and fabrication of a nano patch electrode for ECG using CNT/PDMS,” in *2017 International Conference on Computing Methodologies and Communication (ICCMC)*; July 18–19, 2017; 335–339.

[ref87] Tyack P. L. Johnson M. Aguilar de Soto N. Sturlese A. Madsen P. T. (2006). Extreme diving of beaked whales. J. Exp. Biol. 209, 4238–4253. 10.1242/jeb.02505, PMID: 17050839

[ref88] Weimerskirch H. Guionnet T. Martin J. Shaffer S. A. Costa D. P. (2000). Fast and fuel efficient? Optimal use of wind by flying albatrosses. Proc. R. Soc. London B 267, 1869–1874. 10.1098/rspb.2000.1223, PMID: 11052538 PMC1690761

[ref89] White P. D. King R. L. Jenks J. (1953). The relation of heart size to the time intervals of the heart beat, with particular reference to the elephant and the whale. N. Engl. J. Med. 248, 69–70. 10.1056/NEJM195301082480207, PMID: 13002686

[ref90] Williams T. M. Blackwell S. B. Richter B. Sinding M.-H. S. Heide-Jørgensen M. P. (2017). Paradoxical escape responses by narwhals (*Monodon monoceros*). Science 358, 1328–1331. 10.1126/science.aao2740, PMID: 29217577

[ref91] Williams C. L. Czapanskiy M. F. John J. S. St Leger J. Scadeng M. Ponganis P. J. (2021a). Cervical air sac oxygen profiles in diving emperor penguins: parabronchial ventilation and the respiratory oxygen store. J. Exp. Biol. 224:jeb230219. 10.1242/jeb.230219, PMID: 33257430

[ref92] Williams T. M. Davis R. W. (2021). Physiological resiliency in diving mammals: insights on hypoxia protection using the Krogh principle to understand COVID-19 symptoms. Comp. Biochem. Physiol. A Mol. Integr. Physiol. 253:110849. 10.1016/j.cbpa.2020.110849, PMID: 33227435 PMC8711794

[ref93] Williams T. M. Fuiman L. A. Kendall T. Berry P. Richter B. Noren S. R.. (2015). Exercise at depth alters bradycardia and incidence of cardiac anomalies in deep-diving marine mammals. Nat. Commun. 6:6055. 10.1038/ncomms7055, PMID: 25592286

[ref94] Williams C. L. Hicks J. W. (2016). Continuous arterial P_O2_ profiles in unrestrained, undisturbed aquatic turtles during routine behaviors. J. Exp. Biol. 219, 3616–3625. 10.1242/jeb.141010, PMID: 27618860 PMC5117195

[ref95] Williams C. L. Hindle A. (2021). Field physiology: studying organismal function in the natural environment. Compr. Physiol. 11, 1979–2015. 10.1002/cphy.c200005, PMID: 34190338

[ref96] Williams C. L. Meir J. U. Ponganis P. J. (2011). What triggers the aerobic dive limit? Muscle oxygen depletion during dives of emperor penguins. J. Exp. Biol. 214, 1801–1812. 10.1242/jeb.052233, PMID: 21562166 PMC3092726

[ref97] Williams C. L. Ponganis P. J. (2021). Diving physiology of marine mammals and seabirds: development of biologging techniques. Philos. Trans. R. Soc. B 376:20200211. 10.1098/rstb.2020.0211, PMID: 34121464 PMC8200650

[ref98] Williams C. L. Sato K. Ponganis P. J. (2019). Activity, not submergence, explains diving heart rates of captive loggerhead sea turtles. J. Exp. Biol. 222:jeb200824. 10.1242/jeb.206714, PMID: 30936271

[ref99] Williams C. L. Sato K. Shiomi K. Ponganis P. J. (2012). Muscle energy stores and stroke rates of emperor penguins: implications for muscle metabolism and dive performance. Physiol. Biochem. Zool. 85, 120–133. 10.1086/664698, PMID: 22418705 PMC4887153

[ref100] Williams H. Shipley J. R. Rutz C. Wikelski M. Wilkes M. Hawkes L. (2021b). Future trends in measuring physiology in free-living animals. Philos. Trans. R. Soc. B 376:20200230. 10.1098/rstb.2020.0230, PMID: 34176330 PMC8237165

[ref101] Wright A. K. Ponganis K. V. McDonald B. I. Ponganis P. J. (2014). Heart rates of emperor penguins diving at sea: implications for oxygen store management. Mar. Ecol. Prog. Ser. 496, 85–98. 10.3354/meps10592

